# Machine Learning Algorithms to Detect Sex in Myocardial Perfusion Imaging

**DOI:** 10.3389/fcvm.2021.741679

**Published:** 2021-10-29

**Authors:** Erito Marques de Souza Filho, Fernando de Amorim Fernandes, Maria Gabriela Ribeiro Portela, Pedro Heliodoro Newlands, Lucas Nunes Dalbonio de Carvalho, Tadeu Francisco dos Santos, Alair Augusto Sarmet M. D. dos Santos, Evandro Tinoco Mesquita, Flávio Luiz Seixas, Claudio Tinoco Mesquita, Ronaldo Altenburg Gismondi

**Affiliations:** ^1^Post-graduation in Cardiovascular Sciences, Universidade Federal Fluminense, Niterói, Brazil; ^2^Department of Languages and Technologies, Universidade Federal Rural Do Rio de Janeiro, Rio de Janeiro, Brazil; ^3^Department of Nuclear Medicine, Hospital Universitário Antônio Pedro, Universidade Federal Fluminense, Niterói, Brazil; ^4^Department of Psychology, Hospital Pró-Cardíaco, Rio de Janeiro, Brazil; ^5^Department of Education, Instituto Nacional de Cardiologia, Rio de Janeiro, Brazil; ^6^Institute of Computing, Universidade Federal Fluminense, Niterói, Brazil

**Keywords:** machine learning, myocardial perfusion imaging, sex, general data protection regulation, health care

## Abstract

Myocardial perfusion imaging (MPI) is an essential tool used to diagnose and manage patients with suspected or known coronary artery disease. Additionally, the General Data Protection Regulation (GDPR) represents a milestone about individuals' data security concerns. On the other hand, Machine Learning (ML) has had several applications in the most diverse knowledge areas. It is conceived as a technology with huge potential to revolutionize health care. In this context, we developed ML models to evaluate their ability to distinguish an individual's sex from MPI assessment. We used 260 polar maps (140 men/120 women) to train ML algorithms from a database of patients referred to a university hospital for clinically indicated MPI from January 2016 to December 2018. We tested 07 different ML models, namely, Classification and Regression Tree (CART), Naive Bayes (NB), K-Nearest Neighbors (KNN), Support Vector Machine (SVM), Adaptive Boosting (AB), Random Forests (RF) and, Gradient Boosting (GB). We used a cross-validation strategy. Our work demonstrated that ML algorithms could perform well in assessing the sex of patients undergoing myocardial scintigraphy exams. All the models had accuracy greater than 82%. However, only SVM achieved 90%. KNN, RF, AB, GB had, respectively, 88, 86, 85, 83%. Accuracy standard deviation was lower in KNN, AB, and RF (0.06). SVM and RF had had the best area under the receiver operating characteristic curve (0.93), followed by GB (0.92), KNN (0.91), AB, and NB (0.9). SVM and AB achieved the best precision. Our results bring some challenges regarding the autonomy of patients who wish to keep sex information confidential and certainly add greater complexity to the debate about what data should be considered sensitive to the light of the GDPR.

## Introduction

Myocardial perfusion imaging (MPI) is an essential tool for diagnosing and managing patients with suspected or known coronary artery disease (1). Machine Learning (ML) has had several applications in the most diverse areas of knowledge and is conceived as a technology with huge potential to revolutionize health care (2, 3). We found several applications for the diagnosis (4–6), prognosis (7, 8), and treatment of diseases (9, 10). In particular, the imaging area has significantly benefited from its fruits (11–13). In this context, we highlight at least seven challenges about AI: misuse, increased literacy, the need to use data collected and processed appropriately (healthy data), security, care management based on data, dealing with errors, and the need for cooperation (14). About the concern about the safety of individuals' data, the publication of the General Data Protection Regulation (GDPR) represents a milestone (15). However, this does not exempt the appearance of diverse normative questions, such as issues related to conditions of trustworthiness of ML systems, transparency, explicability, and responsibility (16). Article 9 of the GDPR provides for the processing of particular data. Except in exceptional situations provided for by law, GDPR states that data processing is prohibited in the case of: “personal data revealing racial or ethnic origin, political opinions, religious or philosophical beliefs, or trade union membership, and the processing of genetic data, biometric data to uniquely identify a natural person, data concerning health or data concerning a natural person's sex life or sexual orientation” (15). It is worth to note that sex was not mentioned in the referred GDPR article. In this context, we developed ML models to evaluate their ability to distinguish an individual's sex from assessing myocardial perfusion scintigraphy images. The next section presents an example of a clinical case inserted in the debate, followed by the methods used in this work, the results obtained, discussion, and conclusions.

## Case Example

Consider the following hypothetical example. A male at 18 years old, was successfully submitted to a sex change surgery, having changed his name (AST) and started to sign LMT. Perceiving some people's prejudice regarding her situation, she moved to another city. No one knows about her past in the new city, and she chose to keep this information with her again to avoid falling victim to malicious comments and prejudices, and even violent conduct. At the age of 30, she felt chest pain during a soccer game, having had an electrocardiogram and ultra-sensitive troponin, both without changes. An echocardiogram was performed and showed the diagnosis of hypertrophic cardiomyopathy. Days later, she was referred for myocardial perfusion scintigraphy to assess cardiac function. The patient omitted the sex-change surgery in all the consultations she performed. What to say when an ML algorithm can reveal this information?

## Materials and Methods

**Figure 1:**
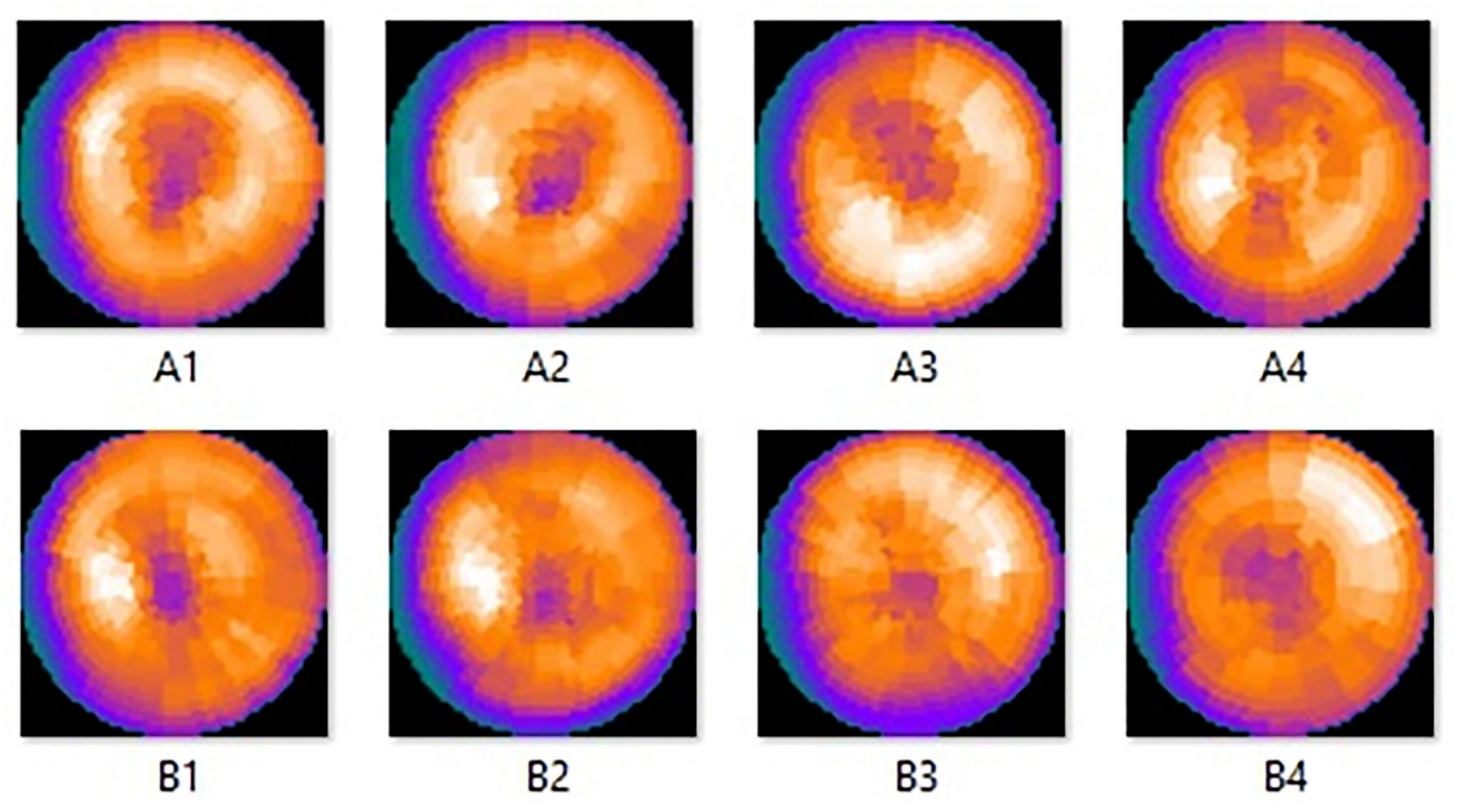
Examples of female polar maps (A1, A2, A3 and A4) and male polar maps (B1, B2, B3 and B4).

Our study is a single-retrospective center, designed to test whether ML algorithms can correctly discern the sex of myocardial perfusion polar maps in the stress and rest position. All images were anonymized and processed by the same physician (EMSF), and the evaluation report indicated an abnormality in all of them. Sex information was obtained from a.xml file associated with each image. A second expert (CTM) reviewed all processes. We used 260 polar maps (140 men/120 women) to train ML algorithms from a database of patients referred to a university hospital for clinically indicated MPI in the period of January 2016 to December 2018—all of them exported from a GE Healthcare Xeleris® workstation in.tiff format and size 175 × 175 (Figure 1).

**Figure 2:**
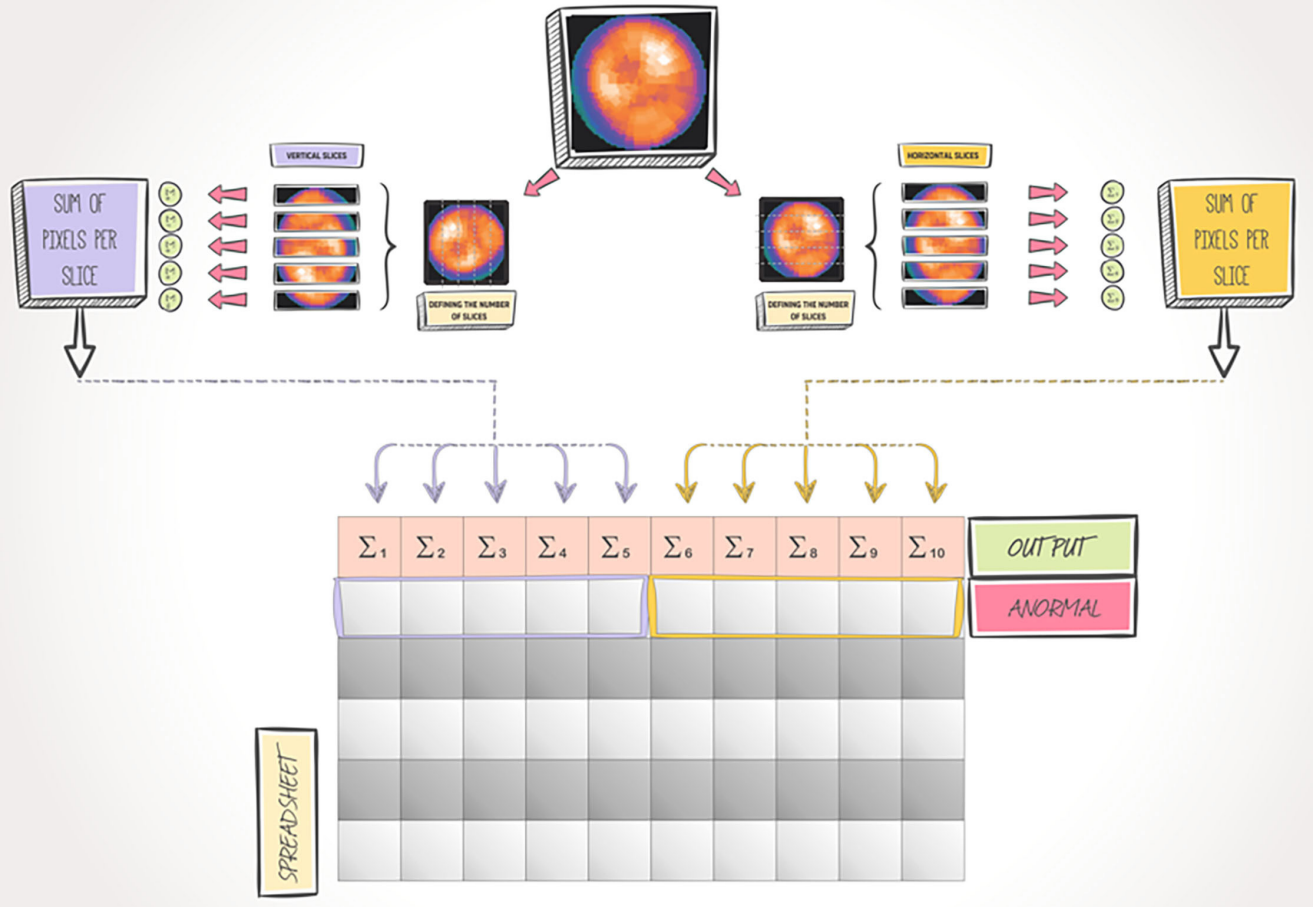
Feature extraction process.

We didn't consider the prone position and clinical information (except sex). Universidade Federal Fluminense's Ethics Committee (in Brazil) approved our work in agreement with the Declaration of Helsinki. In line with ASNC guidelines (17), all images were obtained from patients that performed ECG-gated 2-day Tc-99 m sestamibi myocardial perfusion single-detector SPECT with R-R signal separated in eight-frame, in rest and stress conditions, and supine position having a total acquisition time of 21 min and 64 projections in a 180° orbit. Rest-stress doses were calculated based on the patients' body weight. Ordered-subsets Expectation Maximization (OSEM) algorithm (04 subsets, 10 iterations, and a uniform initial estimate) was used to reconstruct the transaxial emission image (18). Emory Cardiac ToolboxTM (Emory University/ Syntermed, Atlanta, GA) was used for reconstruction, generation, and axis orientation of polar maps. We tested 07 different ML models, namely, Classification and Regression Tree (CART) (19, 20), Naive Bayes (NB) (21, 22), K-Nearest Neighbors (KNN) (23, 24), Support Vector Machine (SVM) (25, 26), Adaptive Boosting (AB) (27, 28), Random Forests (RF) (29, 30), and, Gradient Boosting (GB) (31–33). To better assess the model's predictive capacity, the cross-validation strategy (34, 35) (with k = 10) was used. Sensibility (recall), positive predictive value (precision), F1 measure, and area under the receiver operating characteristic curve (AUC) were used to evaluate the model's performance. We used an image slicing process to obtain image features based on Ouali et al. (36). After generating the polar maps, we implemented an algorithm to acquire information about the intensity of each pixel in the image. Then, we divided each image into 5 horizontal slices and 5 vertical slices (Figure 2). For each generated slice, we calculated the sum of the intensities of each pixel that composes it. After that, we analyzed 10 attributes related to each summing. As it is a supervised learning process, each image was also associated with a label indicating the sex of the patient who selected it. Therefore, we obtained a matrix with 11 columns per 260 rows (one row for each image). The first 10 columns represent the features we used, and the last one corresponds to the label indicating whether the polar map is for a female or male patient. We implemented all ML algorithms in Python 3 using open-source libraries (37, 38). We describe the settings for each ML model in Supplementary Material 2. In our work, only images from myocardial perfusion were used. The Ethics Committee (Universidade Federal Fluminense) has authorized us to use these images as long as they are anonymized (approval number 91399418.2.0000.5243).

## Results

In Table 1, we can see that all the models had accuracy greater than 82%. However, only SVM achieved 90%. KNN, RF, AB, GB had, respectively, 88, 86, 85, 83%. Accuracy standard deviation was lower in KNN, AB, and RF (0.06). SVM and RF had had the best AUC (0.93), followed by GB (0.92), KNN (0.91), AB and NB (0.9). F1 measure ranged from 77% (CART) to 89% (SVM) while precision ranged from 79% (CART) to 86% (SVM, AB). SVM and KNN had the best recall (93%) and CART the worst (80%). SVM and AB achieved the best precision. Recall standard deviation, precision standard deviation, and F1 standard deviation was lower, respectively, in SVM (0.06), KNN (0.8), KNN, and AB (0.06). All computational time was lesser than 2 seconds.

**Table 1:** Computational results.

	**AUC**	**SD**	**Accuracy**	**SD**	**Recall**	**SD**
**CART**	0.77	0.04	0.76	0.07	0.8	0.11
**NB**	0.9	0.07	0.82	0.1	0.82	0.12
**KNN**	0.91	0.05	0.88	0.06	0.93	0.08
**SVM**	0.93	0.05	0.9	0.07	0.93	0.06
**AB**	0.9	0.06	0.85	0.06	0.83	0.07
**GB**	0.92	0.05	0.83	0.07	0.81	0.08
**RF**	0.93	0.04	0.86	0.06	0.87	0.09
	**Precision**	**SD**	**F1**	**SD**		
**CART**	0.79	0.09	0.77	0.07		
**NB**	0.81	0.14	0.81	0.1		
**KNN**	0.84	0.08	0.88	0.06		
**SVM**	0.86	0.09	0.89	0.07		
**AB**	0.86	0.1	0.84	0.06		
**GB**	0.83	0.09	0.82	0.07		
**RF**	0.83	0.09	0.83	0.07		

*CART, Classification and Regression Tree; NB, Naive Bayes; KNN, K-Nearest Neighboors; SVM, Support Vector Machine; AB, Adaptive Boosting; RF, Random Forests; GB, Gradient Boosting; AUC, area under the receiver operating characteristic curve; F1, F1 measure; SD, Standard Deviation; Remark, All computational times were < 02 sec*.

## Discussion

ML algorithms had a high performance to distinguish an individual's sex from myocardial perfusion polar maps. Computational times were very low (<2 seconds), and the store size of images was small because each polar map is less than 25 KB. Three models obtained AUC higher than 90% and had precision, F1, and recall >80%. However, SVM had the best performance of all.

This technique has already been successfully applied in several applications, including as the following: 1—the lung ventilation heterogeneity prediction in patients with chronic obstructive pulmonary disease (AUC: 82% and accuracy: 88%) (12); 2—ventricular arrhythmia prediction using heart rate variability in patients with implantable cardioverter defibrillators (AUC: 0.81 for a 5-min prediction) (39); 3—the assessment of the presence of Alzheimer's diagnosis from functional magnetic resonance images (accuracy: 94.44%) (40).

Besides, it is necessary to understand the differentiation between the aspects mentioned in the example case. Sex is related to biological characteristics, and gender is not limited to defining the difference between the sexes (male and female), emerging as a category of analysis of historical, political, and social processes, presented as a way of reflecting on the relationships established with and between bodies (41). Also, returning to the previous hypothetical clinical case, it is essential to highlight the patient's real intention of not divulging information about her sex, mainly due to the prejudice previously suffered in the city where she used to live. This conduct, it is worth commenting, is the very exercise of human rights of Personality Recognition and Free Development of Personality (42). The fact is that after the assumption of a new gender identity, using sex-change surgery, the patient started to self-determine and to affirm herself through a new personality, which assured her the definitive dignity of her human person (article 1) (42). In this context, information about his/her biological sex has become strange and unnecessary data for his/her new condition of physical-existential life. Besides, it has come to represent a risk to his/her life and personal security (article 3) (42), if improperly revealed, it could be considered as a unique species of iatrogenesis. For example, in Brazil, from 2000 to 2019, there were 4809 violent deaths of people victims of prejudice and intolerance (43). We agree with Stucky et al. (44) on the need for effective measures to reduce discrimination and stigma toward not normative individuals. It is also important to highlight that algorithms can also be used as a prejudice-inducing element. In particular, our algorithm could be useful to uncover information that patients do not want to divulge. Some people may minimize the importance of a situation like this. However, it is worth mentioning that discrimination (in its most diverse types) is an emerging risk factor for various health outcomes (45).

On the other hand, it is essential to point out that this sex information can also be predicted from 12-lead electrocardiogram images, as shown by Attia et al. (46). They trained convolutional neural networks with just under 500,000 images to assess whether this ML model could predict patients' sex. The results were excellent (accuracy: 90% and AUC: 0.97). Therefore, the patient in the clinical case could have the information about her sex revealed in at least two different situations, already known. It is safe to assume that the near future could still demonstrate other techniques to achieve the same end. Wang and Kosinski used neural networks to show that information in a person's photograph contains relevant information, allowing these ML models to recognize the sexual orientation of gays and lesbians. From the analysis of 5 images of each person, the algorithm obtained an accuracy of 91% for distinguishing heterosexual men from gay men and 83% for determining heterosexual women from lesbians (47). In another work, Kosinski demonstrated that facial recognition algorithms could also assess people's political orientation from realistic facial images. The accuracy obtained was 72% (48). In this context, Gilles et al. contrasted mathematical-computational tools and the traditional understanding of medical images as pictures only for visual interpretation (49). These tools can potentially enable a data mining process that allows bringing out hidden information in images—capable of helping the decision-making process and bringing significant harm to people depending on how they are used. The evaluation of image pixels using ML is quite different from that of the human visual cortex, which is much more complex. Still, the results obtained by these kinds of models can be relevant.

Another important point related to the case concerns that the patient possibly did not imagine that this type of processing of her data would be possible. This raises some crucial questions since the amount of different processing that can be performed can be very large. The signing of the free and informed consent form is mandatory (article 6(1)(a) of GDPR) (15). However, this document seems insufficient in some situations, given the possibility that the consent given by the patient may not be, in fact, free and clear, a hypothesis that would violate the principle of transparency (article 5(1)(a) of GDPR) (15), and would contaminate its legal validity. In this context, methodologies for assessing performance, ethical impacts, and privacy play an essential role, such as the tool built by Di Iorio et al. (50) to evaluate data controllers' compliance from computerized survey.

Besides, as reiterated by Mann et al. (51), blockchain can also be useful in this process due to its ability to add security to the entire data processing treatment flow (article 4(2) of GDPR) (15) referring to the MPI. Using blockchain technologies, we can track the data flow since its generation by scintigraphy equipment, passing through by a file located in mobile, network units, or even in the cloud, after being processed by a computer-aided diagnosis software, until its storage and corresponding data. All the precautions above are associated with the Purpose Limitation principle (article 4(3) and article 5(1)(b) of GDPR) (15), whereby states that all the subjects involved with the MPI treatment have to be restricted to the purpose initially established for carrying out such an examination, that is, within the limits of what was previously freely and informed by the patient.

However, the challenges can improve justice, autonomy, and beneficence, bringing trust and patient engagement (51). This position reinforces the importance of attention to the 4P proposed by Garrafa and Porto (52), as it highlights the relevance of taking into account prudence with the unknown, the prevention of possible damage, the precaution in the face of the indiscriminate use of new technologies and the protection of socially excluded, more fragile and vulnerable. Regarding the use of AI in health, the challenges are in the attention to social, political, economic, and ethical impacts, highlighting the importance of caution due to the potential impacts on life and the potential interferences in individual liberties (53).

Within this whole context, however, it is essential to point out the fact that the GDPR does not expressly consider sex information to be sensitive, although our ML models demonstrate a sui generis situation in which the patient's sex deserves the same level of protection guaranteed by article 9 of GDPR (15). Regarding the text of this article, it is essential to highlight Hermeneutics' role in its analysis. First, we must reflect on whether it was written to contain an exhaustive or non-exhaustive list of special categories of personal data. Also, we must reflect on whether such categories can be interpreted extensively or only restrictively. In some cases, it seems that sex and gender identity should be included as one of the special categories of data protection for the present case. The list is non-exhaustive and because of its extensive interpretation. We are talking about protecting human rights and the risk that people will be exposed to a scenario conducive to the spread of prejudice, hate doctrines, persecution, cowardice, and violence. Our point of view rests on the fact that the positivist legal dimension has been overcome since the twentieth century, in such a way that the eminently literal interpretation of the positive norms gave rise to new ideas such as “Critical Rationalism” (54), the “Scientific Revolution” (55), the “Theory of Fundamental Rights” (56), the “Moral Hermeneutics” (57) and many others that make up a new legal philosophy of Post-Positivist and Neoconstitutionalist, through which the positive norm deserves an interpretation that goes beyond its simple literality. It is also important to mention that the Brazilian constitutional court, in recent judgments, determined that issues related to prejudice based on gender identity are comparable to those of racial origin, thus enjoying the same protection.

We are not saying here that the patient should be encouraged to omit his information or lie about it—sex is a variable that could be kept in consideration for investigating several diseases and collected routinely as part of the electronic health record—but we bring an important issue related to his legal right to do so if he deems it necessary and also linked to the awareness of all those who in any way participate in the treatment of data related to this type of exam. Considering this panorama, it is also essential to emphasize the role of the physician-patient relationship. When the patient perceives it as trustworthy and helpful, we have an excellent foundation to get the right information and that the treatment is followed (58). Still, within this debate, there are the corresponding challenges linked to all those who participate in the treatment of data related to the exam. They must be responsible for any treatment that goes beyond what was freely and clarified informed by the patient and answer for the occurrence of any personal data breach (article 4(12) of GDPR)(15). Therefore, considering this panorama, it is worth highlighting the concerns of Sun et al.: the fear that digital technologies may, in fact, result in human rights violations is real and is based on people's experiences about social marginalization, surveillance and discrimination (59) which brings up the necessity of discerning how to apply the existing human-rights framework to new digital technologies (60) and the need for humans to be in the decision-making process (61). In this context, artificial intelligence could expand the human capacity to cast its gaze on transhuman aspects providing this reality to overcome the limits imposed by gaze (62). However, its use linked to technological determinism can cause complex impacts for its applicability in human ways of living.

Our study has clear limitations. Despite the promising results obtained, the number of images is small. In addition to that, it is a retrospective study performed at a single medical center. On the other hand, the discussion focused on the GDPR, leaving aside other laws worldwide. However, this text raises a critical topic under discussion and several questions not yet fully clarified. Therefore, future works should consider expanding the number of polar maps for training and testing the models in multicenter studies. Also, the use of other legal sources can contribute to discussions on this topic.

## Conclusion

Our work demonstrated that ML algorithms could perform well in assessing the sex of patients undergoing myocardial scintigraphy exams. Thus, this brings some challenges regarding the autonomy of patients who wish to keep this information confidential and certainly adds greater complexity to the debate about what data should be considered sensitive to the light of the GDPR.

## Data Availability Statement

The original contributions presented in the study are included in the article/Supplementary Material, further inquiries can be directed to the corresponding author/s.

## Author Contributions

ES, FF, CM, and RG: conception or design of the work. ES, FF, TS, and CM: data collection. ES, FS, and LC: data analysis. ES, FS, CM, RG, MP, and PN: interpretation. ES, FF, MP, PN, FS, CM, and RG: drafting the article. AS and EM: critical revision of the article. ES, FS, CM, and RG: final approval of the version to be published. All authors contributed to the article and approved the submitted version.

## Conflict of Interest

The authors declare that the research was conducted in the absence of any commercial or financial relationships that could be construed as a potential conflict of interest.

## Publisher's Note

All claims expressed in this article are solely those of the authors and do not necessarily represent those of their affiliated organizations, or those of the publisher, the editors and the reviewers. Any product that may be evaluated in this article, or claim that may be made by its manufacturer, is not guaranteed or endorsed by the publisher.
