# Supplementary material for: Machine Learning Algorithms to Detect Sex in Myocardial Perfusion Imaging
Source: Front Cardiovasc Med. 2021 Oct 29;8:741679. doi: 10.3389/fcvm.2021.741679 (PMC8585770; doi:10.3389/fcvm.2021.741679)
Supplement: Supplementary file 2 [file Table_2.DOCX]

**Supplementary Data 2 (Table B): Machine Learning settings**

|  | **CART** | **NB** | **KNN** | **SVM** | **AB** | **GB** | **RF** |
| --- | --- | --- | --- | --- | --- | --- | --- |
| bootstrap | na | na | na | na | na | na | True |
| ccp_alpha | 0.0 | na | na | na | na | 0.0 | 0.0 |
| class_weight | None | na | na | None | na | na | None |
| criterion | Gini | na | na | na | na | friedman_mse | Gini |
| splitter | Best | na | na | na | na | na | na |
| max_depth | None | na | na | na | na | 3 | None |
| max_features | None | na | na | na | na | None | auto |
| max_leaf_nodes | None | na | na | na | na | None | None |
| max_samples | na | na | na | na | na | na | None |
| min_impurity_decrease | 0.0 | na | na | na | na | 0.0 | 0.0 |
| min_impurity_split | 0 | na | na | na | na | None | None |
| min_samples_leaf | 1 | na | na | na | na | 1 | 1 |
| min_samples_split | 2 | na | na | na | na | 2 | 2 |
| min_weight_fraction_leaf | 0.0 | na | na | na | na | 0.0 | 0.0 |
| n_estimators | na | na | na | na | 50 | 100 | 100 |
| n_jobs | na | na | na | na | na | na | None |
| oob_score | na | na | na | na | na | na | False |
| random_state | None | na | na | None | None | None | None |
| verbose | na | na | na | False | na | 0 | 0 |
| warm_start | na | na | na | na | na | False | False |
| init | na | na | na | na | na | None | na |
| learning_rate | na | na | na | na | 1.0 | 0.1 | na |
| loss | na | na | na | na | na | deviance | na |
| n_iter_no_change | na | na | na | na | na | None | na |
| presort | na | na | na | na | na | deprecated | na |
| subsample | na | na | na | na | na | 1.0 | na |
| tol | na | na | na | 0.0001 | na | 0.0001 | na |
| validation_fraction | na | na | na | na | na | 0.1 | na |
| algorithm | na | na | ‘auto’ | na | SAMME.R | na | na |
| base_estimator | na | na | na | na | None | na | na |
| priors | na | None | Na | na | na | na | na |
| var_smoothing | na | 1e-10 | na | na | na | na | na |
| n_neighbors | na | na | 1 to 17 | na | na | na | na |
| weights | na | na | ‘uniform’ | na | na | na | na |
| leaf_size | na | na | 30 | na | na | na | na |
| p | Na | Na | 2 | na | na | na | na |
| metric | Na | Na | ‘Minkowski’ | na | na | na | na |
| metric_params | Na | Na | None | na | na | na | na |
| n_jobs | na | na | None | na | na | na | na |
| C | na | na | na | 1.0 | na | na | na |
| kernel | na | na | na | ‘rbf’ | na | na | na |
| degree | na | na | na | 3 | na | na | na |
| gamma | na | na | na | ‘auto’ | na | na | na |
| coef0 | na | na | na | 0.0 | na | na | na |
| shrinking | na | na | na | True | na | na | na |
| probability | na | na | na | False | na | na | na |
| cache_size | na | na | na | 200 | na | na | na |
| max_iter | na | na | na | -1 | na | na | na |
| decision_function_shape | na | na | na | ‘ovr’ | na | na | na |

na: not applicable. We have implemented all algorithms in Python. Scikit-learn and other libraries also were used. CART: Classification and Regression Trees. NB: Naive Bayes. KNN: k-nearest neighbors. SVM: Support Vector Machine. AB: AdaBoost. GB: Gradient Boosting. RF: Random Forests.
